# Gestational age‐related changes in relaxation times of neonatal brain by quantitative synthetic magnetic resonance imaging

**DOI:** 10.1002/brb3.3068

**Published:** 2023-05-29

**Authors:** Yan Dong, Xianyu Deng, Meizhen Xie, Lan Yu, Long Qian, Ge Chen, Yali Zhang, Yanyun Tang, Zhipeng Zhou, Liling Long

**Affiliations:** ^1^ Department of Radiology, The First Affiliated Hospital of Guangxi Medical University Nanning China; ^2^ Department of Radiology, Affiliated Hospital of Guilin Medical University Guilin Medical University Guilin China; ^3^ Department of Cardiovascular Guilin People's Hospital Guilin China; ^4^ Department of Biomedical Engineering College of Engineering Peking University Beijing China

**Keywords:** linear regression analysis, newborn, quantification of relaxation time, synthetic MRI

## Abstract

**Objective:**

This study aimed to explore the correlation between T1 and T2 relaxation times of synthetic MRI (SyMRI) and gestational age (GA) in each hemisphere of preterm and term newborns at the initial 28 days of birth.

**Methods:**

Seventy preterm and full‐term infants were prospectively included in this study. All subjects completed 3.0 T routine MRI and SyMRI (MAGiC) one‐stop scanning within 28 days of birth (aged 34–42 W at examination). The SyMRI postprocessing software (v8.0.4) was used to measure the T1 and T2 relaxation values of each brain region. The linear regression equations of quantitative relaxation values with GA were established to compare the variation speed in each brain region.

**Results:**

A significant linear and negative correlation was found between relaxation times and GA in the neonate cerebral cortex and subcortical gray and white matter regions (All *p*<.05). The relaxation time of the left centrum semiovale decreased with maximum variance with increasing GA among all white matter regions (T1: *b* = –51.45, *β* = –0.65, *p* < .0001; T2: *b* = –8.77, *β* = –0.71, *p* < .0001), whereas the right posterior limb of internal capsule showed minimal variance (T1: *b* = –27.94, *β* = –0.60, *p* < .0001; T2: *b* = –3.25, *β* = –0.68, *p* < .0001). Among all gray matter regions, the right globus pallidus and thalamus indicated the most significant decreasing degree of T1 and T2 relaxation values with GA (right globus pallidus T1: *b* = –33.14, *β* = –0.64, *p* < .0001; right thalamus T2: *b* = –3.94, *β* = –0.81, *p* < .0001), and the right and left occipital lobes indicated the least significant decreasing degree of T1 and T2 relaxation values with GA, respectively (right occipital lobes T1: *b* = –11.18, *β* = –0.26, *p* = .028; left occipital lobes T2: *b* = –1.22, *β* = –0.27, *p* = .024).

**Conclusions:**

SyMRI could quantitatively evaluate the linear changes of T1 and T2 relaxation values with GA in brain gray and white matter of preterm and term neonates.

## Graphical abstract

1. SyMRI could quantitatively evaluate the linear changes of T1 and T2 relaxation values with GA in brain gray and white matter of preterm and term neonates. 2. The relaxation times of white matter indicated faster‐decreasing trends with the increasing GA than those of gray matter. 3. The relaxation times of gray and white matters were asymmetrical between the left and right cerebral hemispheres with GA.

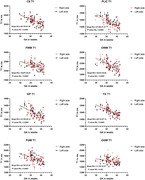

## 1 INTRODUCTION

Preterm birth is a global health problem and the leading cause of death in children under 5 years of age (Liu et al., 2016), associated with more than 30,000 infants yearly, second only to congenital abnormalities in China (He et al., 2017). Preterm birth is also the most common cause of chronic neurological diseases due to cerebral palsy and neurobehavioral disorders (Back, 2017). Considering the complexity and rapid development of the neonatal brain, the evaluation of neonatal brain structure and maturity may be the key to detecting early preterm brain injury and subsequent neurodevelopmental disorders for timely treatment and surveillance (Fenchel et al., 2020; Holland et al., 2014; Huppi et al., 1998).

Magnetic resonance imaging (MRI) is a valuable tool for neuroimaging evaluation and predicting neonatal motor and cognitive function using quantitative approaches (de Vries et al., 2015; Fenchel et al., 2020; Huppi et al., 1998). In recent years, synthetic MRI (SyMRI) has emerged with significant advantages in quantitative T1, T2, and proton density (PD) values (Hagiwara et al., 2017; Lee et al., 2018) rapid imaging and postprocessing (Hagiwara et al., 2017), and robust assessment of brain volume (Kim et al., 2022).

SyMRI is essentially a single‐period, multicontrast imaging technology. SyMRI is much faster than the multiperiod conventional MRI (Liu et al., 2021). A 6‐min scan can obtain quantitative imaging of the relaxation rate covering the whole brain and complete anatomical imaging, whereas conventional MRI brain sequence acquisition takes 20–60 min (Hagiwara et al., 2017). In addition, we can obtain multicontrast images in about 1 min using SyMRI software (Hagiwara et al., 2019).

Relaxation time has been demonstrated to correlate with neonatal brain maturity (Caiwen et al., 2021; Kim et al., 2022). The previous studies have explored the correlation between SyMRI relaxation time and gestational age (GA) in premature infants or children with the assumption of bilateral brain symmetry (Caiwen et al., 2021; McAllister et al., 2017; Vanderhasselt et al., 2021). However, there are rare cases of bilateral brain symmetry in anatomy, function, and gene expression (Dehaene‐Lambertz et al., 2006; Dehaene‐Lambertz et al., 2002; Erberich et al., 2006; Gilmore et al., 2007; Lin et al., 2013; Mahmoudzadeh et al., 2013; McCartney & Hepper, 1999; Sun & Walsh, 2006; Sun et al., 2005). Anatomically, the volume of the left hemisphere of a newborn is larger than that of the right hemisphere (McAllister et al., 2017). On the functional level, early hearing and language processing of infants have left brain advantages (Dehaene‐Lambertz et al., 2006; Gilmore et al., 2007). Asymmetry of frontal lobe activation has been observed at the sixth month of gestation (Dehaene‐Lambertz et al., 2002). Somatosensory response and movement lateralization can be detected at birth or even in the second trimester of pregnancy (Erberich et al., 2006; Mahmoudzadeh et al., 2013). In clinical work, some diseases occur specifically on one side. For example, primary progressive aphasia is characterized by the neurodegeneration of the left cerebral cortex (Mesulam et al., 2022). The lateralization of unilateral Parkinson's disease symptoms corresponds to differences in neuronal molecular biology (Li et al., 2020). Common cerebral hemispheres infarction on both sides of the human brain and bilateral subcortical vascular lesions have asymmetrical effects on attention (Fimm et al., 2001). Current studies have shown that this asymmetry may be closely related to the differences in the internal structure of neurons and tissues on both sides of the brain and the asymmetry of gene expression (Galaburda et al., 1978; Li et al., 2020; Sun & Walsh, 2006). To our knowledge, no one has reported the correlation between the T1 and T2 values of SyMRI and GA in preterm and term neonates from the respective left and right sides of the brain.

This study aimed to explore the correlation between T1 and T2 relaxation times of SyMRI and GA at the initial 28 days of birth in each hemisphere of preterm and term newborns.

## 2 METHODS

### 2.1 Patients

**FIGURE 1:**
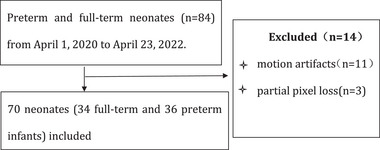
Flowchart of preterm and full‐term neonates.

We prospectively included preterm and full‐term neonates admitted to the Department of Neonatology, Affiliated Hospital of Guilin Medical College, who underwent head MRI examinations between April 1, 2020, and April 23, 2022. Patients were excluded if they showed one of the following three criteria: (1) the presence of severe complications, including bilirubin encephalopathy, congenital infections, congenital metabolic diseases, craniocerebral malformations, and other organ injuries; (2) the age of MRI examination more than 28 days; (3) obvious motion artifacts or partial pixel loss in SyMRI images. A total of 84 neonates with appropriate weight for gestational age were included initially, in which 11 were excluded due to motion artifacts and 3 were excluded due to partial pixel loss. Ultimately, 70 neonates (34 full‐term and 36 preterm infants) were included in the study (Figure 1). Demographic characteristics, including sex, GA, postmenstrual age (PMA), and birth weight (BW), were included in the study (Table 1). The PMA was calculated based on the sum of GA and age at the MR examination.

**TABLE 1:** Demographic parameters and clinical characteristics.

	Total number (*n* = 70)
**Quantitative variable**	
GA (week) PMA (week)	37.08 ± 2.76 37.64 (36.43, 40.14)
Birth weight (kg)	2.66 ± 0.68
**Categorical variable**	
Sex (female∖male)	22/48

The present prospective study was approved by the Ethics Committee of our hospital (Approval number: 2022WJWZCLL‐15) with the informed consent of the newborn's guardian before the MRI examination.

### 2.2 MRI examination

All neonates were sedated by enema (10% chloral hydrate 0.5 mL/kg) or intramuscular administration of phenobarbital (10 mg/kg) 30 min before the MRI examination for assurance of quiet and hearing protection in neonates.

Head MRI scans were performed on a GE SIGNATM Architect 3.0T device using a 48‐channel phased front coil. Conventional scans, including axial T2WI PROPELLER, T1WI‐FLAIR (fluid‐attenuated inversion recovery), T2WI‐FLAIR, DWI, sagittal T1WI‐FLAIR, and diffusion‐weighted imaging (DWI), were performed, followed by MAGiC transverse axis sequence scans. The specific magnetic resonance acquisition parameters of this study are shown in Table 2.

**TABLE 2:** Magnetic resonance imaging acquisition parameters.

	Axial SyMRI	Axial T2WI PROPELLER	Axial T1‐weighted fluid‐attenuated inversion recovery (FLAIR)	Axial T2‐weighted fluid‐attenuated inversion recovery (FLAIR)	Axial diffusion‐weighted imaging (DWI)	Sag‐T1‐weighted fluid‐attenuated inversion recovery (FLAIR)
Matrix	288×288	320×320	320×224	256×256	160×160	320×224
Field of view (cm^2^)	180×180	180×180	180×135	180×135	180×135	160×160
Slice thickness (mm)	4	4	4	4	4	4
Spacing (mm)	0.4	0.4	0.4	0.4	0.4	1.0
TR (ms)	4364.0	4922.0	1750.0	4574.0	4574.0	1750.0
TE (ms)	21.8	100.1	24	145.0	90.3	24.0
Pixel size	0.6×0.6×4	0.6×0.6×4	0.6×0.8×4	0.7×0.7×4	1.1×0.8×4	0.5×0.7×4
Number of sections	20	20	20	20	20	15
Acquisition time (min)	5:32	1:24	1:35	1:57	1:02	1:51

### 2.3 Measurement of quantitative relaxation values in SyMRI

SyMRI sequence is a multilayer, multiecho, and multisaturation delay saturation recovery spin‐echo (SE) acquisition method. The algorithm first uses multiple TE images to determine the T2 relaxation time. Then, images with different saturation delay times (TD) are used to obtain the first estimates of T1 and PD through the exponential fitting of the equation (Warntjes et al., 2008). Finally, T1 and PD are recalculated and refined using the locally effective rollover angles of saturated and excited radio frequency (RF) pulses (Krauss et al., 2015).

Data were imported into GE SIGNATM Architect 3.0T host workstation for postprocessing (Figures 6 and 7). In this study, each brain hemisphere had eight regions of interest (ROIs), including centrum semiovale (CS), posterior limb of internal capsule (PLIC), frontal white matter (FWM), occipital gray matter (OGM), frontal gray matte (FGM), occipital gray matter (OGM), globus pallidus (GP), and thalamus (TH). Each newborn brain had a total of 16 ROIs. The left and right sides were abbreviated as L and R, respectively; for example, the left CS was present as LCS, and the right CS was present as RCS. Two hemispheres were manually depicted on the T1WI sequence of SyMRI for each subject (Figures 4 and 5). All ROIs were drawn by the first author (Yan Dong) of this manuscript. The rules of ROIs are as follows: keep size symmetry on both sides of the same anatomical region as far as possible. After avoiding edges and sulci, the ROI size was selected as the maximum range delineated in the corresponding anatomical area at the selected level. Each ROI was measured three times, and the final value was the average of the three measurements.

### 2.4 Statistical methods

The statistical analysis was completed using IBM SPSS Statistics 26.0. A *p* value less than 0.05 indicated statistical significance. In this study, PMA was present with nonnormal distribution, and the distribution of BW and GA was evaluated with normality. The distribution of relaxation time variables was evaluated with normality or approximate normality that T2 values of right GP (RGP) and right TH (RTH) and T1 values of left OGM (LOGM) were present with approximately normal distribution. The remaining 13 ROIs on T1 and T2 values were normally distributed data. Single‐factor linear regression was used to explore the relationship between T1 and T2 relaxation times and GA in each hemisphere, and slope (regression coefficient *b*) was used to evaluate the speed of variation in quantitative relaxation value with GA for each ROI. The *t* test is the statistical method used to test the significance of regression coefficient values.

## 3 RESULTS

### 3.1 Correlation between quantitative relaxation times and neonatal clinical characteristics

The T1 and T2 values of each region were significantly correlated with GA, PMA, and BW (all < .05). GA indicated the strongest correlation with relaxation time among all clinical characteristics. After the adjustment of BW, relaxation time was still significantly correlated with GA in each ROI, whereas it was not significantly correlated with BW after the adjustment of GA. There was no significant correlation between sex and relaxation time (*p* > .05).

### 3.2 Single‐factor linear regression analysis between quantitative relaxation time and GA

The decreasing speed of T1 and T2 values was ranked with the increasing GA in each ROI as follows (Table 3):

**TABLE 3:** Linear regression of synthetic MRI relaxation time T1, T2, and GA in neonatal brain regions.

		T1				T2			
ROI	Side	Adj‐*R* ^2^	*b*	*β*	*p* Value	Adj‐*R* ^2^	*b*	*β*	*p* Value
CS	R	0.35	−46.19	−0.60	<.0001	0.46	−7.83	−0.68	<.0001
	L	0.41	−51.45	−0.65	<.0001	0.50	−8.77	−0.71	<.0001
PLIC	R	0.35	−27.94	−0.60	<.0001	0.45	−3.25	−0.68	<.0001
	L	0.43	−31.37	−0.66	<.0001	0.47	−3.28	−0.69	<.0001
GP	R	0.40	−33.14	−0.64	<.0001	0.52	−3.32	−0.73	<.0001
	L	0.37	−30.14	−0.61	<.0001	0.49	−3.28	−0.71	<.0001
TH	R	0.45	−29.13	−0.68	<.0001	0.65	−3.94	−0.81	<.0001
	L	0.47	−27.14	−0.69	<.0001	0.62	−3.59	−0.79	<.0001
FWM	R	0.26	−36.87	−0.52	<.0001	0.35	−6.51	−0.60	<.0001
	L	0.20	−32.51	−0.46	<.0001	0.32	−7.13	−0.58	<.0001
FGM	R	0.17	−22.78	−0.42	<.0001	0.16	−1.96	−0.42	<.0001
	L	0.27	−27.57	−0.53	<.0001	0.32	−2.22	−0.57	<.0001
OWM	R	0.38	−39.51	−0.63	<.0001	0.38	−6.52	−0.62	<.0001
	L	0.31	−34.54	−0.57	<.0001	0.35	−6.58	−0.60	<.0001
OGM	R	0.06	−11.18	−0.26	.028	0.10	−1.28	−0.33	.005
	L	0.09	−12.37	−0.32	.007	0.06	−1.22	−0.27	.024

T1 values for each white matter (WM) region: LCS>RCS>ROWM>RFWM>LOWM>LFWM>LPLIC>RPLIC;

T2 values for each WM region: LCS>RCS>LFWM>LOWM>ROWM>RFWM>LPLIC>RPLIC;

T1 values for each gray matter (GM) region: RGP>LGP>RTH>LFGM>LTH>RFGM>LOGM>ROGM;

T2 values for each GM region: RTH>LTH>RGP>LGP>LFGM>RFGM>ROGM>LOGM.

The residuals of the univariate regression models between relaxation times and GA in all ROIs were present with normality, variance homogeneity, and independence.

## 4 DISCUSSION

In the present study, we determined that SyMRI could linearly and quantitatively evaluate the associations between relaxation time and GA in both preterm and term neonates, which might be used to evaluate the brain maturity and development of white and gray matter.

In this study, gender was not found to significantly influence the quantitative relaxation time of each hemisphere in neonates, which was consistent with the quantitative relaxation study on SyMRI technology in the adult human brain (Hagiwara et al., 2021).

The accuracy and repeatability of quantitative measurements of relaxation time and PD value of brain tissue by SyMRI have been confirmed in previous studies (Krauss et al., 2015; Vanderhasselt et al., 2021; Vanderhasselt et al., 2020).

We found that T1 and T2 relaxation times of WM decreased faster than those of GM as GA increased, which might be attributed to a large amount of myelination in white matter during the neonatal period (Korogi et al., 1996; Morel et al., 2021).

**FIGURE 2:**
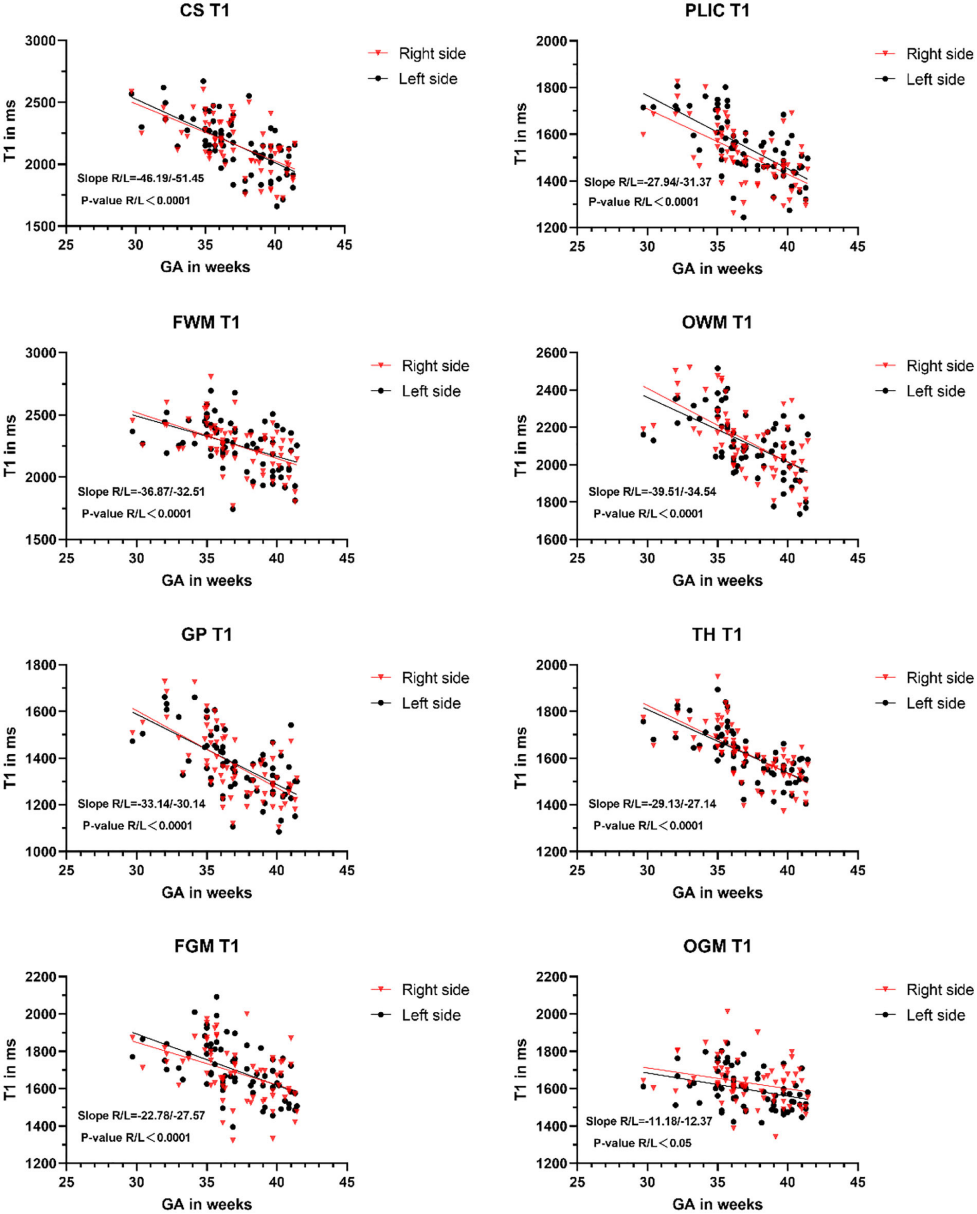
Linear regression relationship between T1 value and GA in each brain region of the left and right sides of neonates (slope in ms/week).

**FIGURE 3:**
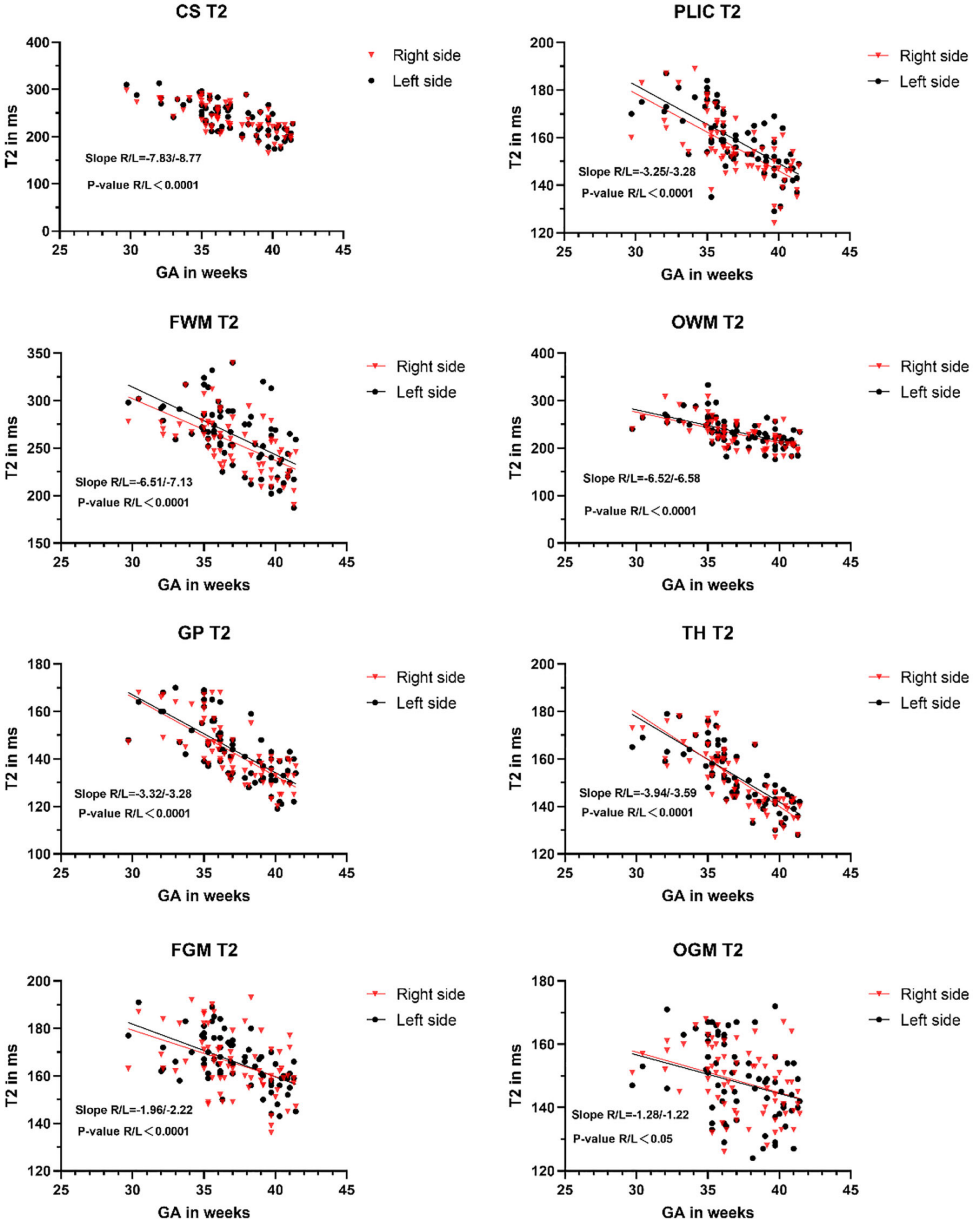
Linear regression relationship between T2 value and GA in each brain region of the left and right sides of neonates (slope in ms/week).

Among all white matter ROIs, the relaxation time of LCS decreased maximally with the increasing GA, whereas RPLIC decreased minimally with the increasing GA (Figures 2 and 3 ). These findings were inconsistent with the study led by Vanderhasselt et al. (2021), which found the obvious significance of PLIC.

**FIGURE 4:**
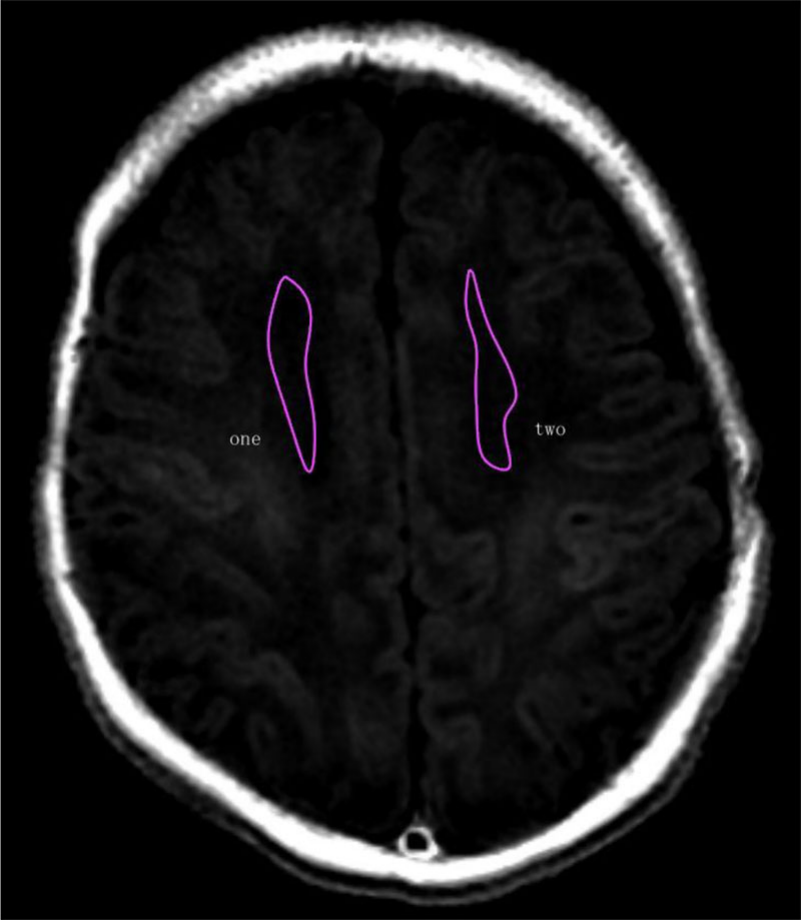
Schematic diagram of ROI selection (MAGiC T1 FLAIR, axial view of centrum semiovale).

**FIGURE 5:**
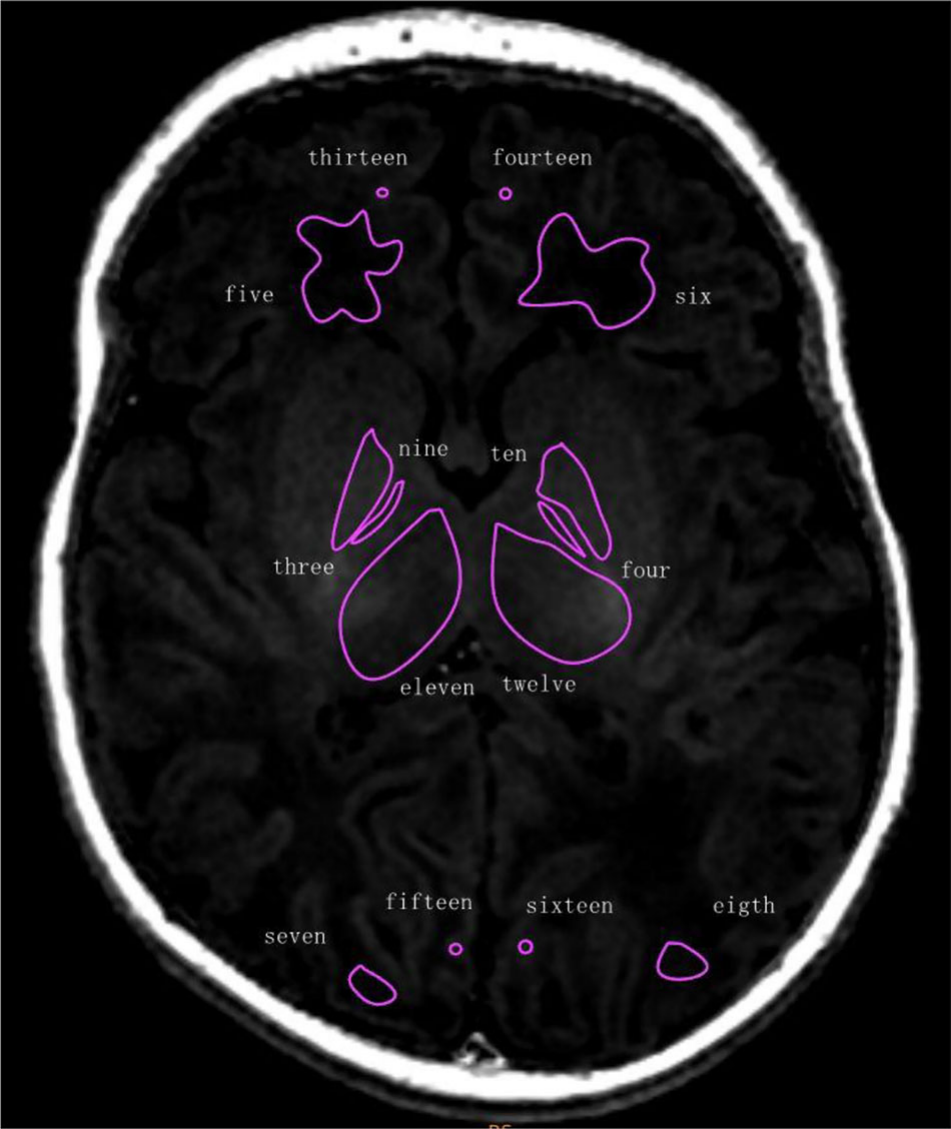
Schematic diagram of ROI selection (MAGiC T1 FLAIR, axial view of basal ganglia). White matter region (1–8): 1, 2: centrum semiovale, CS (Figure 4); 3, 4: posterior limb of internal capsule, PLIC; 5, 6: frontal white matter, FWM; 7, 8: occipital white matter, OWM. Gray matter region (9–16): 13, 14: frontal gray matte, FGM; 15, 16: occipital gray matter, OGM; 9, 10: globus pallidus, GP; 11, 12: thalamus, TH.

**FIGURE 6:**
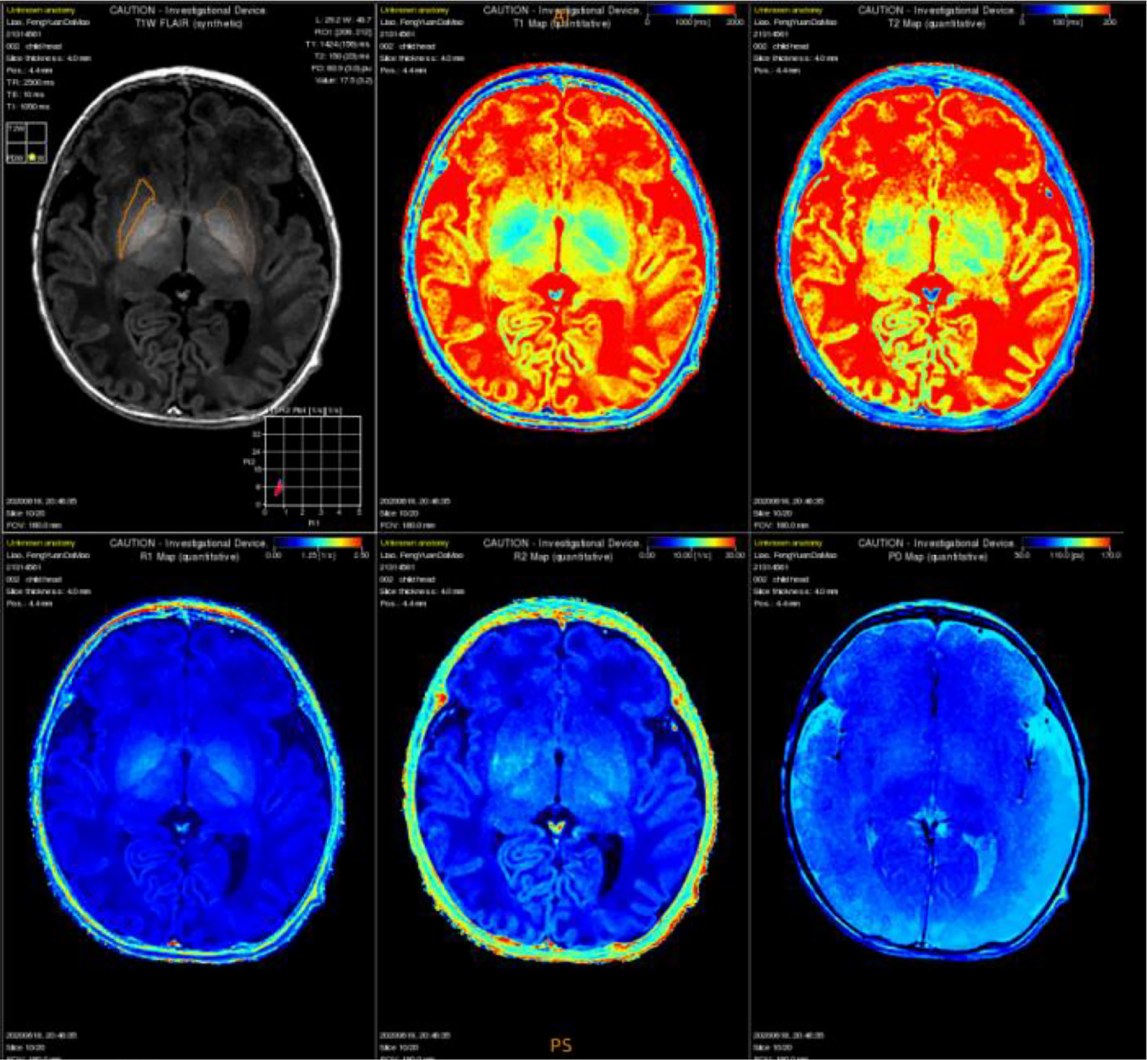
Quantitative ROI (globus pallidus) mapping and T1 mapping, T2 mapping, R1 mapping, R2 mapping, and PD mapping diagram of one‐term MAGiC T1 FLAIR sequence at basal ganglia level (first figure, first row) obtained by SyMRI scan.

**FIGURE 7:**
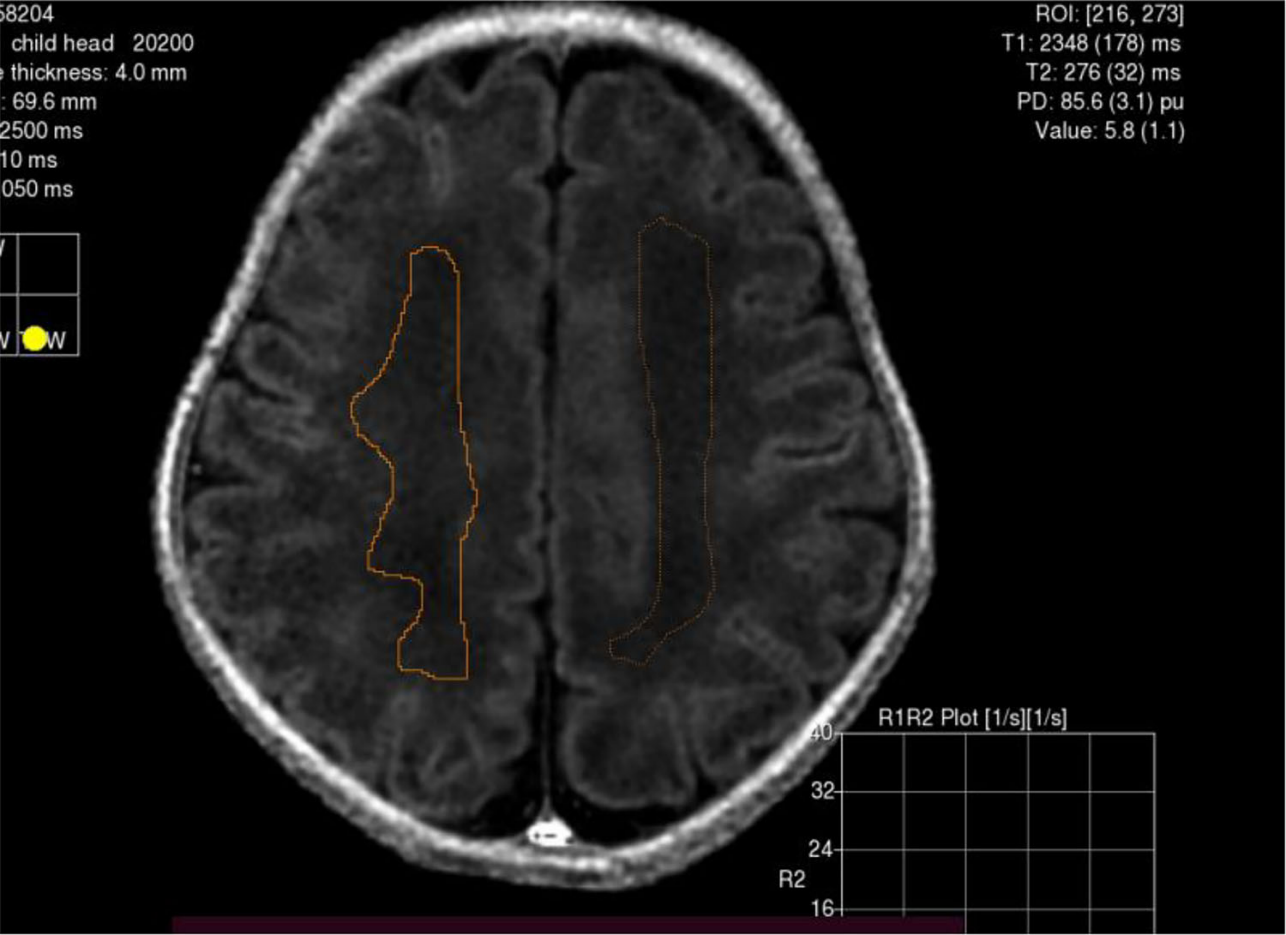
Quantitative relaxation time (T1: 2348 ± 178 ms, T2: 276 ± 32 ms) of the right centrum semiovale region of a preterm newborn were measured on MAGiC T1 FLAIR sequence.

During the first 3 years of life, the increasing anisotropy in nondense white matter structures of CS has been proven to be significantly greater than that in dense white matter structures of PLIC (McGraw1 et al., 2002). However, the rate of myelination in nondense white matter structures has been demonstrated faster histologically compared with dense white matter structures (Brody et al., 1987; Kinney et al., 1988).

The relaxation time of neonatal CS rather than PLIC decreased significantly in a linear dependence on GA, which might be related to the faster myelination rate of nondense white matter structures in the neonatal period. Vanderhasselt et al. (2021) also pointed out no correlation between relaxation time in the frontal lobe, parietal lobe, and central semiovale with corrected GA. Nevertheless, the T1 and T2 values in all ROIs, including the frontal lobe, occipital lobe, and central semiovale, were correlated with GA significantly in the present study (all *p* < .05), which might have been induced by different GA periods selected in the two studies.

The decreasing T1 value in gray matter with GA was determined with obvious significance in RGP (Figure 2) as the region with the highest nonheme iron content in all ROIs with the property of shortening T1 (Hallgren & Sourander, 1958).

The most significant decreasing T2 value in gray matter with GA was found in RTH (Figure 3), and the linear regression model between thalamic T2 value and GA was identified with the strongest correlation among all ROIs (RTH: Adj‐*R* ^2^ = 0.65, LTH: Adj‐*R* ^2^ = 0.62, all *p* < .001), which implied that thalamic T2 value was easily affected by GA in the neonatal period. Although GP and TH belong to deep subcortical gray matter nuclei, abundant white matter tracts remain inside (Qiu et al., 2013). Nearly one‐third of the internal white matter tracts in GP are myelinated at birth (Melbourne et al., 2016), and the myelination process generates and enhances the internal connections between subcortical gray matter and its connections with the cortex (Qiu et al., 2013).

The relaxation time of subcortical gray matter (GP and TH) decreased faster with the increasing GA than that of frontal and occipital gray matter (Figures 2 and 3). The theory about myelination could not fully explain the variation of relaxation time in cortical and subcortical gray matter with GA. There might be internal microstructural changes caused by the late development of nerve cells, proliferation of axons, and glia (Korogi et al., 1996; Melbourne et al., 2016), and even changes in calcium and iron deposition (Raz et al., 2003), which formed the combined action to decrease relaxation time rapidly (Tullo et al., 2019).

Although the iron content in each brain region of the newborn is lower than that of the adult (Hallgren & Sourander, 1958), the present study found that the relaxation time of each brain region in the newborn changed with the speed of GA. The distribution of nonheme iron content in the adult brain, for example, the iron content in the core of the extrapyramidal system was higher than that in the frontal and occipital gray matter of the cerebral cortex, and the concentration of nonheme iron in white matter was higher than that in the gray matter (Hallgren & Sourander, 1958). In this study, the correlation between left and right occipital gray matter relaxation times and GA showed the minimum significance among all ROIs. The changes of T1 and T2 values in the gray matter of the frontal lobe were greater than those in the gray matter of the occipital lobe with GA (Figures 2 and 3), which might be attributed to the rapid maturity of the occipital lobe compared with the frontal lobe (Gilmore et al., 2007) inducting slight variance of relaxation time with GA. This finding was consistent with the previous results that SyMRI relaxation in the occipital lobe of the adult brain remains relatively stable with age, but the frontal lobe changes obviously with age (Badve et al., 2015).

The results of relaxation time variation with GA in the deep subcortical gray matter on both sides indicated that the right side seemed more significant than the left. However, the deep subcortical white matter results contradicted it (Figures 2 and 3). These findings were per the well‐accepted rule that the volume of the subcortical gray matter region was asymmetric to the right, and that of the subcortical white matter region was asymmetric to the left (Dean et al., 2018). It suggested that the different changing rates of relaxation time with GA in subcortical structures between the left and right hemispheres supported the existence of asymmetry in subcortical gray and white matter during the neonatal period, which was consistent with the trend of volume asymmetry. Previous studies about cortical asymmetry have confirmed gene expression asymmetry in the embryonic cortex at the third month of human pregnancy (Sun & Walsh, 2006). It was still unknown whether there is asymmetric gene expression in human subcortical structure asymmetry based on the findings about asymmetric neural coding in the guinea pig auditory cortex on both sides of the hypothalamus in previous animal experiments (King et al., 1999).

The present study found that the right side represented more obvious T1 changes with GA in the frontal and occipital white matter than on the left side (Figure 2), where the corresponding T2 value was more evident on the left side (Figure 3). However, there was no clear left and right dominance with GA in the relaxation time of the frontal and occipital gray matter. On the one hand, there might be more obvious asymmetry in the gray matter for frontal and occipital lobes than white matter (Good et al., 2001). On the other hand, a weaker correlation between the relaxation time of occipital gray matter and GA was found compared with that between white matter and GA (adj‐*R* ^2^ of occipital gray matter was the smallest in all ROIs).

### 4.1 Limitations

First, SyMRI images are too sensitive to generate motion artifacts. Second, the data from the cerebellum, brainstem, and corpus callosum fluctuated clearly with skewed distribution due to the anatomical location and volume effect, which was not included in the present analysis. In addition, even the latest SyMRI 11.1 segmentation algorithm could not achieve accurate distinguishing performance between GM and WM for newborns compared with MANTiS (McAllister et al., 2017). Third, a slice thickness of 4 mm for neonate SyMRI sequences is a bit thick, and ROI measurements for SyMRI are currently available only manually. Fourth, the current study did not apply the promising magnetic resonance fingerprinting technology that could quantify multiple tissue properties (T1, T2, and magnetization transfer) (Hilbert et al., 2020).

Fortunately, relaxation values in each ROI were linearly and negatively correlated with GA, which was almost consistent with the results of the latest research that found that T1 relaxation value was linearly correlated with GA in the newborn brain by quantifying regional differences and maturation of the newborn brain using a 3D magnetic resonance fingerprint (Yu et al., 2022). In addition, we obtained a linear correlation between the T2 value and GA, which was not found by magnetic resonance fingerprinting. Finally, the current study did not evaluate the prognosis of preterm and term neonates. Our research team is identifying the influence of relaxation value on the prognosis of preterm and term neonates.

In conclusion, the linear changes in T1 and T2 relaxation values of neonatal brain SyMRI related to GA reflected the differences and asymmetric differences in the maturity of bilateral cortical and subcortical gray/white matter. It could assist in the early assessment of neonatal neural development and cognitive abnormalities by evaluating the agreement between the relaxation time of each region and GA, considering the linear relationship between the relaxation time of the neonatal brain and GA.

### 4.2 Moral recognition

This study was approved by the Ethics Committee of the hospital (Approval number: 2022WJWZCLL‐15). Informed consent was obtained from the family members of the subjects before the MR examination.

## AUTHOR CONTRIBUTIONS

Yan Dong and Xianyu Deng participated in literature reading, statistical analysis, article conception, and writing. Liling Long guided me in the direction of the project. Zhipeng Zhou and Yanyun Tang gave technical support. Long Qian contributed to postprocessing guidance. Meizhen Xie, Lan Yu, Yali Zhang, and Ge Chen collected original data.

### PEER REVIEW

The peer review history for this article is available at https://publons.com/publon/10.1002/brb3.3068.

## Data Availability

The data supporting the findings of this study are available from the corresponding author upon reasonable request.
